# An Infrared Small Target Detection Method Based on Attention Mechanism

**DOI:** 10.3390/s23208608

**Published:** 2023-10-20

**Authors:** Xiaotian Wang, Ruitao Lu, Haixia Bi, Yuhai Li

**Affiliations:** 1 Unmanned System Research Institute, Northwestern Polytechnical University, Xi’an 710072, China or 18710993786@163.com (X.W.);; 2 School of Information and Communication Engineering, Xi’an Jiaotong University, Xi’an 710049, China; 3 Department of Missile Engineering, Rocket Force University of Engineering, Xi’an 710025, China; 4 National Key Laboratory of Electromagnetic Space Security, Tianjin 300308, China; liyuhai.cn@gmail.com

**Keywords:** infrared small target detection, feature frequency domain fusion, attention mechanism, feature weighting adjustment

## Abstract

The human visual attention system plays an important role in infrared target recognition because it can quickly and accurately recognize infrared small targets and has good scene adaptability. This paper proposes an infrared small target detection method based on an attention mechanism, which consists of three modules: a bottom-up passive attention module, a top-down active attention module, and decision feedback equalization. In the top-down active attention module, given the Gaussian characteristics of infrared small targets, the idea of combining knowledge-experience Gaussian shape features is applied to implement feature extraction, and quaternion cosine transform is performed to achieve multi-dimensional fusion of Gaussian shape features, thereby achieving complementary fusion of multi-dimensional feature information. In the bottom-up passive attention module, considering that the difference in contrast and motion between the target and the background can attract attention easily, an optimal fast local contrast algorithm and improved circular pipeline filtering are adopted to find candidate target regions. Meanwhile, the multi-scale Laplacian of the Gaussian filter is adopted to estimate the optimal size of the infrared small target. The fast local contrast algorithm based on box filter acceleration and structure optimization is employed to extract local contrast features, and candidate target regions can be obtained by using an adaptive threshold. Besides, the mean gray, target size, Gaussian consistency, and circular region constraint are used in pipeline filtering to extract motion regions, and the false-alarm rate is reduced effectively. Finally, decision feedback equalization is adopted to obtain real targets. Experiments are conducted on some real infrared images involving complex backgrounds with sea, sky, and ground clutters, and the experimental results indicate that the proposed method can achieve better detection performance than conventional baseline methods, such as RLCM, ILCM, PQFT, MPCM, and ADMD. Also, mathematical proofs are provided to validate the proposed method.

## 1. Introduction

Detecting and tracking infrared small targets has been a challenging and extensively studied topic in the military field. This technique is commonly used in infrared search and tracking systems, infrared weapon guidance, and missile early warning systems [1,2,3]. In infrared scenes, small targets are often surrounded by strong background clutter and noise, while the limited imaging area and lack of distinct features such as shape and texture make detection and tracking infrared small targets difficult at a long imaging distance. Especially for backgrounds with sea, sky, and ground clutters, the detection difficulty further increases due to dynamic changes, low signal-to-noise ratio, fluctuations in target energy, and even submergence. The imaging characteristics and complex and diverse backgrounds of the aforementioned small infrared targets pose great challenges to the current detection task. Therefore, it is urgent to study how to detect infrared small targets accurately, robustly, and in real-time [4,5,6,7].

The primary methods of small infrared target detection can be categorized as filtering-based methods, low-rank sparse restoration-based methods, and feature continuity-based methods. The filtering-based method exploits the difference between the target and background clutter in the spatial and frequency domains to detect the target. Various filters have been introduced, including the adaptive toggle operator [8], nonnegativity-constrained variational mode decomposition [9], and frequency-tuned salient region detection [10]. These methods have a small amount of calculation, but the detection effect is poor. They can only suppress the uniform background to a certain extent, and the suppression effect is poor in complex backgrounds with sea, sky, and ground clutters. The low-rank sparse restoration-based method utilizes the difference in frequency characteristics of the target and the background clutter to detect the target [11,12,13]. However, the large number of calculations limit the engineering application of this type of method. The feature continuity-based method fully utilizes the temporal continuity of the shape and gray level of the target, as well as prior information such as the continuity of the motion trajectory to distinguish the target from the background clutter [14,15,16]. This type of method has a good detection effect for fast-moving targets, but it will fail for stationary targets. Therefore, the existing methods cannot be used in engineering practice to realize accurate, robust, and real-time detection in complex backgrounds.

In recent years, the human visual attention system has been introduced into the research of infrared small target detection. The human visual attention system can be divided into two types: the data-driven bottom-up passive attention mechanism and the task-dependent top-down active attention mechanism. Although infrared small targets are very weak, there are certain differences between the targets and the local background, and the human visual attention system can capture these differences and locate these areas quickly. Inspired by active attention and passive attention mechanisms, researchers have innovatively proposed various algorithms and made great breakthroughs [17,18,19,20].

By imitating the human visual attention mechanism, local contrast is adopted to effectively enhance the target area and suppress background clutter and noise, such as LCM (Local Contrast Measure) [21], ILCM (Improved Local Contrast Measure) [22], LDAM (Local Difference Adaptive Measure) [23], RLCM (Relative LCM) [24], DLCM (Double-Layer Local Contrast Measure) [25], MLHM (Multiscale Local Homogeneity Measure) [26], LIG (Local Intensity and Gradient) [27], HB-MLCM (High-Boost-based Multiscale Local Contrast Measure) [28], MPCM (Multiscale Patch-based Contrast Measure) [29], WSLCM (Weighted Strengthened Local Contrast Measure) [30] and ADMD (Absolute Directional Mean Difference) [31], etc. Based on strong representational capabilities, many deep learning methods have also begun to be widely used [32,33].

The local contrast-based methods mentioned above only use passive attention information to detect small infrared targets, but ignore active attention information, which can introduce many false alarms. Meanwhile, it is necessary to select features that can accurately distinguish between real targets and false-alarm targets. To overcome the aforementioned limitations, this paper proposes a small target detection method based on an attention mechanism. The proposed method employs passive and active attention information to improve detection performance. The main contributions of this paper are summarized as follows:

(1) A top-down active attention module is proposed to obtain target knowledge-experience Gaussian shape features, and the quaternion cosine transform is used to achieve multi-dimensional fusion of Gaussian shape features, thereby significantly improving the signal-to-clutter ratio gain (SCRG) and background suppression factor (BSF).
(2) The difference in contrast and motion between the target and the background is exploited to design a bottom-up passive attention module; meanwhile, an optimal fast local contrast algorithm and improved circular pipeline filtering are adopted to find candidate target regions, using passive attention features with a discriminative ability to accurately detect small infrared targets.
(3) The decision feedback equalization considers the results of active and passive attention mechanisms to find real targets, which can better adapt to environmental changes in different scenarios.
(4) The remainder of this paper is organized as follows. In Section 2, the related work—such as the attention method, quaternion method, and ILCM—is reviewed. Section 3 presents the general framework of the proposed method and provides an in-depth explanation of its mathematical proofs. Section 4 outlines the experiments to validate the proposed method. Finally, Section 5 concludes this paper.

## 2. Related Work

In this section, the attention method, the quaternion method, and LCM are reviewed in brief, all of which have a strong connection to our research.

### 2.1. Attention Method

**Figure 1:**
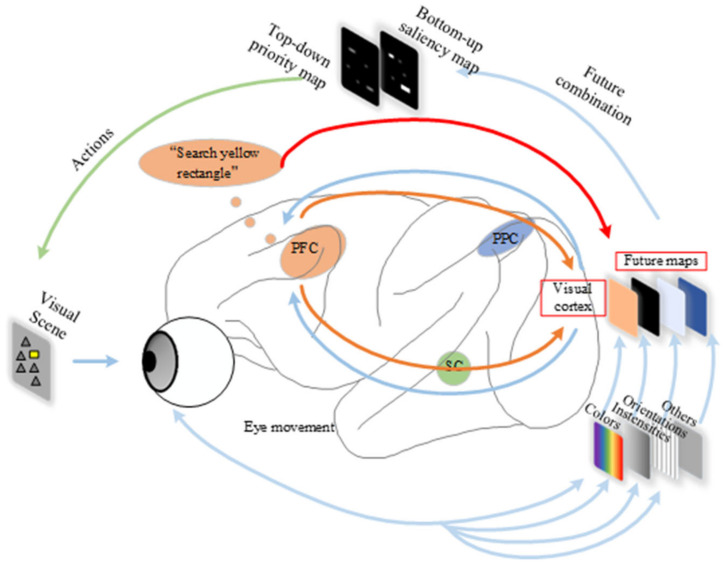
The human visual attention system.

The human visual attention system can be divided into two types: the data-driven bottom-up passive attention mechanism and the task-dependent top-down active attention mechanism, as shown in Figure 1. In the bottom-up passive attention mechanism, the prefrontal cortex (PFC) and posterior parietal cortex (PPC) integrate the original physical characteristics of external stimuli that are transmitted through the visual pathway to form a complete saliency map in the brain. This causes eye movement controlled by the superior colliculus (SC) and makes it easy for us to pay attention to the yellow rectangular target in the visual scene (the task here is to search the yellow rectangle). The physical features here include color, intensities, orientations, etc. The top-down active attention mechanism actively searches for targets with priority map characteristics in the prefrontal cortex (PFC) of the brain according to known task information and knowledge experience; it causes eye movement so that we can easily pay attention to the desired target.

Inspired by the human visual attention system, the simulation and simplification of each attention module can realize the accurate detection of infrared small targets.

### 2.2. Quaternion Method

The quaternion discrete cosine transform (QDCT) algorithm combines discrete cosine transform (DCT) with quaternion algebra, and it is widely used in image processing. Object detection has been realized through input variables by applying QDCT in recent times. The stronger the feature generalization ability, the higher the detection performance of the algorithm.

The quaternion representation is:

(1) $$I_{q}=x_{1}+x_{2}i+x_{3}j+x_{4}k$$

where $x_{1}$, $x_{2}$, $x_{3}$ and $x_{4}$ are four real numbers, and they satisfy the operation rule $i^{2}=j^{2}=k^{2}=ijk=-1,ij=-ji=k,jk=-kj=i,ki=-ik=j$. Each quaternion $I_{q}$ is a linear combination of 1, i, j, and k.

Assume that the resolution of $I_{q}$ is *M×N*, where *M* and *N* represent the length and width of the image, respectively. The QDCT of $I_{q}$ is given by

(2) $$Q(u,v)=QDCT(I_{q})=\alpha_{u}^{M}\alpha_{v}^{N}\sum_{m=0}^{M-1}\sum_{n=0}^{N-1}\mu_{Q}I_{q}N(u,v,m,n)$$

where $\alpha_{u}^{M}$ and $\alpha_{v}^{N}$ are the coefficients. N(u,v,m,n) and $\mu_{Q}$ are given by

(3) $$\left\{\begin{array}{l} N(u,v,m,n)=\cos\left[\frac{\pi }{M}(m+\frac{1}{2})u\right]\cos\left[\frac{\pi }{N}(n+\frac{1}{2})v\right]\\ \mu_{Q}=-\sqrt{\frac{1}{3}i}-\sqrt{\frac{1}{3}j}-\sqrt{\frac{1}{3}k} \end{array}\right.$$

The corresponding inverse quaternion cosine transform (IQDCT) is shown below:

(4) $$I_{q}{}^{\prime }=IODCT(Q(u,v))=\sum_{u=0}^{M-1}\sum_{v=0}^{N-1}\alpha_{u}^{M}\alpha_{v}^{N}\mu_{q}Q(u,v)N(u,v,m,n)$$

The quaternary cosine transform can be employed to fuse the input feature variables to realize the complementary fusion of feature information of different dimensions and achieve target detection.

### 2.3. LCM Algorithm

The traditional LCM algorithm uses a sliding window to traverse the entire image pixel by pixel from left to right and top to bottom to calculate the local contrast of the image, consequently achieving accurate detection of small targets. The sliding window contains 3 × 3 sub-windows in total. Among the sub-windows, the one in the center denoted by “0” represents the target area, and the ones in the surrounding area denoted by “1”, “2”, “3”, “4”, “5”, “6”, “7”, and “8” represent the background area.

**Figure 2:**
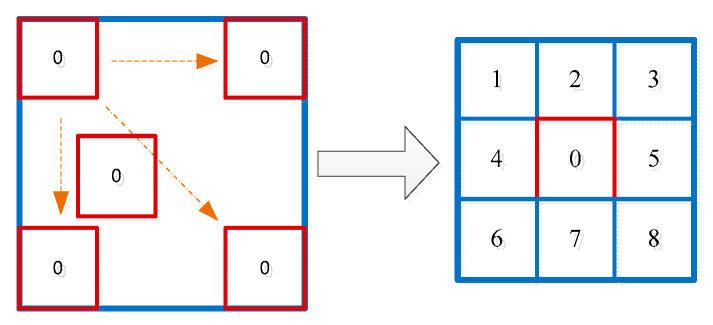
The calculation principle of LCM.

The average gray value of each sub-window can be expressed as

(5) $$m=\frac{1}{N}\sum_{i,j}I_{d}(i,j)$$

where N is the number of pixels in each sub-window, and $I_{d}(i,j)$ is the gray value in the sub-window whose center is located at the coordinates (i,j). When the sliding window passes through a certain pixel of the image, the LCM of the pixel can use the central sub-window and the eight adjacent background sub-windows, and its expression is:

(6) $$C=\min(L_{0}\times m_{0}^{}/m_{k}^{})(k=1,2,\ldots ,8)$$

where $L_{0}$ and $m_{0}^{}$ are the maximum gray value and mean gray value of the sub-window “0”, respectively, while $m_{k}^{}(k=1,2,\ldots ,8)$ represents the mean gray value of the eight surrounding sub-windows. The eight surrounding sub-windows serve as the local background (see Figure 2).

The fast local contrast algorithm based on box filter acceleration and structure optimization is an improved version of the LCM, and it has a faster calculation speed while ensuring accuracy.

## 3. Methodology

**Figure 3:**
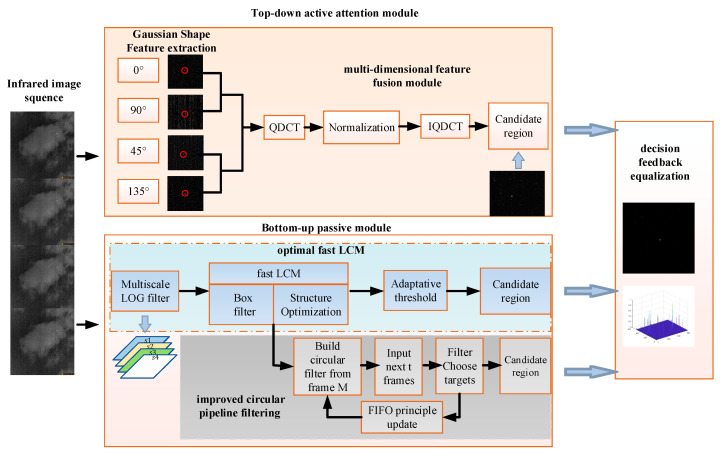
The flowchart of the proposed method.

In the top-down active attention module, to fully utilize the knowledge-experience information of infrared small targets, this paper extracts Gaussian shape features and uses QDCT to achieve complementary fusion of multi-dimensional Gaussian shape features. In the bottom-up passive attention module, the difference in contrast and the motion between the target and the background can attract attention easily, and based on this, this paper proposes an optimal fast local contrast algorithm and uses improved circular pipeline filtering to find candidate target regions, which can reduce the false alarm rate and improve detection precision effectively. Additionally, decision feedback equalization is adopted to detect real targets, and it is effective in different scenarios to better adapt to environmental changes. Figure 3 shows the flowchart of the proposed method, followed by a detailed description of the specific execution steps. The introduction of the top-down active attention module is outlined in Section 3.1, followed by the explanation of the bottom-up passive attention module in Section 3.2. Section 3.3 discusses decision feedback equalization, and Section 3.4 presents the optimal fast local contrast algorithm along with its mathematical proofs.

### 3.1. Top-Down Active Attention Module

**Figure 4:**
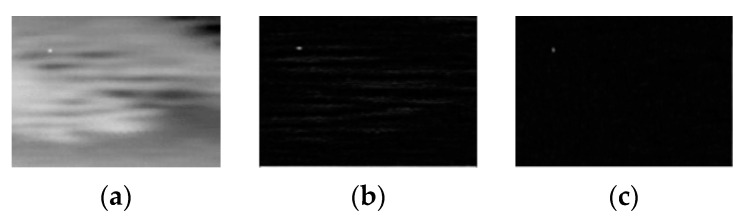
The effect of the second-order directional derivative filter. (**a**) The original image; (**b**) the processing effect in the horizontal direction; (**c**) the processing effect in the vertical direction.

The top-down active attention module exploits the prior knowledge of the infrared small target to design a discriminator and effectively detect the infrared small target. Due to the use of an optical imaging system in Gaussian shape feature extraction, infrared small targets appear as isotropic Gaussian-like spots while the background typically exhibits a uniform direction. To distinguish the Gaussian-like spots and strip-like textures that have a single direction, the second-order directional derivative filter is adopted to construct a continuous function within a specific area using discrete points, and then, the directional derivative of the function is determined. As shown in Figure 4, this method can effectively extract the desired features.

Denote the gray value of the image at the coordinate point (x,y) as f(x,y), and denote the directional derivative $D_{i}$ of the direction vector l as:

(7) $$\begin{array}{cc} D_{i} & =\frac{\partial^{2}f(x,y)}{\partial l^{2}}\left|\begin{array}{l} {}_{\left(x_{0},y_{0}\right)} \end{array}\right.\\ & =\left[f_{xx}(x,y)\cos^{2}\alpha +2f_{xy}(x,y)\cos\alpha \cos\beta +f_{yy}(x,y)\cos^{2}\beta \right]\left|\begin{array}{l} {}_{\left(x_{0},y_{0}\right)} \end{array}\right. \end{array}$$

where α is the angle between the direction vector l and the X-axis (row of image), and β is the angle between the direction vector l and the Y-axis (column of image).

Because the target has the same Gaussian characteristics, the Gaussian-like target is retained in the detection process. Meanwhile, to better suppress the background clutter, four directions $\alpha =0^{\circ },45^{\circ },90^{\circ },135^{\circ }$ are selected for calculation. So, the Gaussian shape features of the target can be obtained

(8) $$\left[D_{1}(x,y),D_{2}(x,y),D_{3}(x,y),D_{4}(x,y)\right]$$

In the multi-dimensional feature fusion module, the quaternion cosine transform is used to achieve complementary fusion of multi-dimensional Gaussian shape features [34]. The Gaussian shape features are represented by $D_{1}(x,y)$, $D_{2}(x,y)$, $D_{3}(x,y)$, and $D_{4}(x,y)$ respectively. They are taken as four data channels to construct quaternion representation:

(9) $$q(x,y)=\lambda_{1}*D_{1}(x,y)+\lambda_{2}*D_{2}(x,y)\vec{i}+\lambda_{3}*D_{3}(x,y)\vec{j}+\lambda_{4}*D_{4}(x,y)\vec{k}$$

where $\vec{i}$,$\vec{j}$, and $\vec{k}$ represent three imaginary axes, and $\lambda_{1},\lambda_{2},\lambda_{3}$, and $\lambda_{4}$ correspond to different data channel weights; here, they are set to 0.15, 0.15, 0.35, and 0.35, respectively.

Assume that the resolution of q(x,y) is *M×N*, where *M* and *N* represent the length and width of the image, respectively. The QDCT of q(x,y) is given by

(10) $$Q(u,v)=QDCT(q(x,y))=\alpha_{u}^{M}\alpha_{v}^{N}\sum_{m=0}^{M-1}\sum_{n=0}^{N-1}\mu_{Q}q(x,y)N(u,v,m,n)$$

where $\alpha_{u}^{M}$ and $\alpha_{v}^{N}$ are the coefficients. N(u,v,m,n) and $\mu_{Q}$ are given by Equation (3).

The normalization function is used for Q(u,v) to suppress the low-frequency information of the background and enhance the high-frequency information of the target. The frequency-domain normalization function is shown below:

(11) $$Q^{\prime }=\mathrm{sgn}(Q)=\left\{\begin{array}{cc} \frac{x_{0}}{\left|Q\right|}+\frac{x_{1}}{\left|Q\right|}i+\frac{x_{2}}{\left|Q\right|}j+\frac{x_{3}}{\left|Q\right|}k & \left|Q\right|\neq 0\\ 0 & \left|Q\right|=0 \end{array}\right.$$

where $x_{0}$, $x_{1}$, $x_{2}$, and $x_{3}$ are four components of Q, respectively. |Q| is the magnitude of the quaternion. Finally, the corresponding IQDCT is used to obtain the spatial domain saliency map as follows:

(12) $$\begin{array}{cc} q^{\prime }(x,y) & =IODCT(\mathrm{sgn}(QDCT(q(x,y))))\\ & =IQDCT(Q^{\prime })\\ & =\sum_{u=0}^{M-1}\sum_{v=0}^{N-1}\alpha_{u}^{M}\alpha_{v}^{N}\mu_{q}Q^{\prime }(u,v)N(u,v,m,n) \end{array}$$

To make the saliency map smoother, the smoothing process is performed by using the Gauss smooth filter to obtain the final map. The formula is as follows:

(13) $$S(x,y)=g(x,y,\sigma =1.5)*\left[q^{\prime }(x,y)⊙{\bar{q}}^{\prime }(x,y)\right]$$

Then, the candidate region of the top-down active attention module can be obtained and denoted as $R_{a}$.

### 3.2. Bottom-Up Passive Attention Module

Based on the contrast and motion characteristics of infrared small targets, the bottom-up passive attention module designs a discriminator to effectively detect targets. An optimal fast local contrast algorithm is proposed in this paper to find the candidate target regions by using contrast characteristics of infrared small targets, and improved circular pipeline filtering is adopted to find candidate target regions using motion characteristics of infrared small targets. The difference in contrast and motion between the target and the background can attract passive attention easily, which is a discriminative characteristic for accurately detecting small infrared targets.

**Figure 5:**
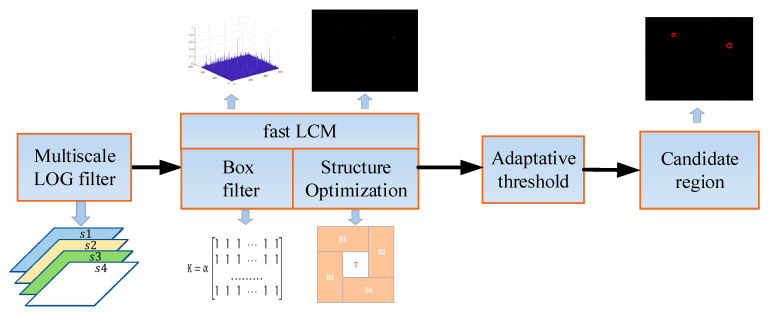
The flowchart of the proposed optimal fast local contrast algorithm.

Since the scale of infrared small targets has a certain range, it is necessary to cover all targets with scales ranging from 2 × 2 to 9 × 9. When contrast feature extraction is performed by calculating the local contrast at multiple scales, the target and clutter background will be enhanced at the same time, and the detection effect is not ideal. To resolve this problem, this paper proposes an optimal fast local contrast algorithm to extract candidate regions. In this algorithm, the multiscale Laplacian of Gaussian (LOG) filter is used to estimate the optimal scale of the infrared small target. Meanwhile, the fast local contrast algorithm based on box filter acceleration and structure optimization is employed to extract local contrast features, and candidate target regions can be obtained by an adaptive threshold. The flowchart of the proposed optimal fast local contrast algorithm is shown in Figure 5.

To obtain the sub-window scale of the corresponding image pixel f(x,y), the LOG filter is used to estimate the sub-window scale in advance and obtain the optimal scale of the infrared small target. The formula of the multiscale LOG filter with a scale-space constant $s_{i}(i=1,2,3,4)$ is shown as follows

(14) $$LoG(x,y,s_{i})=\frac{1}{2\pi s_{i}{}^{2}}\left[\frac{x^{2}+y^{2}-2s_{i}{}^{2}}{s_{i}{}^{4}}\right]e^{\frac{-x^{2}-y^{2}}{2s_{i}{}^{2}}}$$

The scale-space constant for detecting small infrared targets should satisfy the criterion that the size of the target is slightly smaller than $2\sqrt{2}s_{i}$, i.e., the scale-space constant corresponding to small infrared targets (around 3 × 3 pixels) should be 1.1. In this study, the scale of a small target is smaller than 9 × 9 pixels. To guarantee the real-time performance of the algorithm, four typical scales are selected. The scale-space constant $s_{i}$ corresponding to small infrared targets (3 × 3 pixels, 5 × 5 pixels, 7 × 7 pixels, and 9 × 9 pixels) are [1.1 1.77 2.48 3.19]. The optimal sub-window scale is defined as

(15) $$F=\max(F_{i})=LoG(x,y,s_{i})⊗f(x,y)(i=1,2,3,4)$$

**Figure 6:**
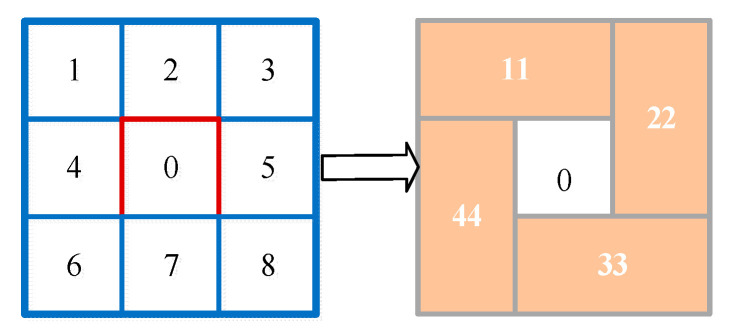
The structure optimization of the fast local contrast algorithm.

At each position, the optimal sub-window scale can be obtained to design the local contrast algorithm. Based on box filter acceleration and structure optimization, a fast local contrast algorithm is proposed in this paper to extract the local contrast features. As shown in Figure 6, the sub-window in the center denoted by “0” represents the target area, and sub-windows in the surrounding area are four rectangular sub-windows denoted by “11”, “22”, “32”, “44”, respectively [3]. The original sub-windows of the LCM algorithm denoted by “1” and “2” are combined into “11”, those denoted by “3” and “5” are combined into “22”, those denoted by “7” and “8” are combined into “33”, and those denoted by “4” and “6” are combined into “44”. The difference $d_{i}$ between the central sub-window “0” and its four neighboring sub-windows with the optimal sub-window scale $\sigma_{i}$ is defined as

(16) $$d_{i}(x,y,\sigma_{i})=\frac{(m_{0}^{\sigma_{i}}-m_{Bi}^{\sigma_{i}}{}_{i})m_{0}^{\sigma_{i}}}{m_{Bi}^{\sigma_{i}}{}_{i}}(i=1,2,\ldots ,4)$$

where $m_{0}^{\sigma_{i}}$ and $m_{Bi}^{\sigma_{i}}{}_{i}$ are the mean gray value of the central sub-window “0” and its four neighbor sub-windows with the optimal sub-window scale $\sigma_{i}$, respectively.

The fast local contrast map with the optimal sub-window scale $\sigma_{i}$ can be defined as

(17) $$S(x,y,\sigma_{i})=\min\{d_{1}*d_{3},d_{2}*d_{4}\}$$

**Figure 7:**
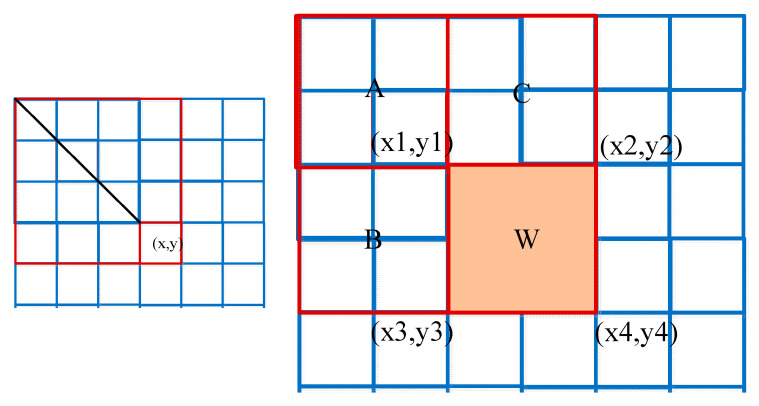
Box filter acceleration.

To accelerate the mean gray calculation of four rectangular neighbor sub-windows, a box filter is introduced. As shown in Figure 7, the box filter mainly converts the gray of the image pixel f(x’,y’) into the sum of the gray value of the corresponding diagonal region from the upper left corner to any point (x,y). Its mathematical formula is

(18) $$I(x,y)=\sum_{x’\leq x,y’\leq y}f(x’,y’)$$

The value at location (x1,y1) is the sum of the pixels in rectangle A. The value at location (x2,y2) is A + C, at location (x3,y3) is A + C, and at location (x4,y4) is A + B + C + W. The sum within W can be computed as I(x1,y1)+I(x4,y4)−I(x2,y2)−I(x3,y3). The mean gray calculation of four rectangular neighbor sub-windows can be converted to simple additions and subtractions as follows

(19) $$\sum W=\frac{I(x1,y1)+I(x4,y4)-I(x2,y2)-I(x3,y3)}{\left(x2-x1)(y4-y2\right)}$$

Therefore, the fast local contrast algorithm based on box filter acceleration and structure optimization can extract the local contrast features. Meanwhile, to detect candidate small infrared targets, an adaptive threshold method is used. The candidate small infrared target region S’(x,y) can be expressed as

(20) $$\left\{\begin{array}{ll} S’(x,y)=S(x,y) & S(x,y)\geq T\\ S’(x,y)=0 & S(x,y)<T\\ T=\mu_{S}+k•\sigma_{S} \end{array}\right.$$

where $\mu_{S}$ and $\sigma_{S}$ denote the mean and variance of the local contrast map S(x,y), respectively; k=3 is the proportionality coefficient. Further, T is the adaptive threshold value.

**Figure 8:**
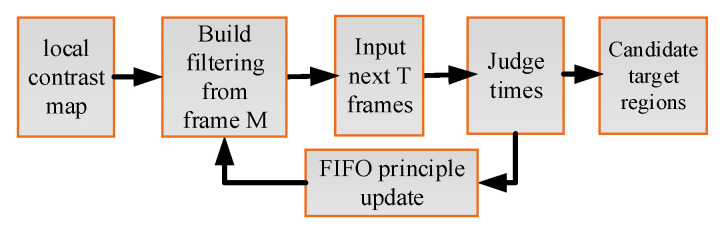
The flowchart of improved circular pipeline filtering.

As shown in Figure 8, to reduce the false alarm rate, improved circular pipeline filtering is adopted to find candidate target regions using motion characteristics of infrared small targets. The mean gray, target size, and circular region constraints are used in pipeline filtering to extract motion regions [35].

To find the moving target areas, the local contrast map obtained by the optimal fast local contrast algorithm is regarded as an input of the pipeline as follows:

(21) $$H’=\{{H’}_{1},{H’}_{2},\ldots ,{H’}_{l}\}$$

where H’ denotes the centers of suspected target patches, and l is the number of local contrast patches.

Concentric ring pipeline filtering with an inner radius $r_{1}$ and an outer radius $r_{2}$ is set, and the step sizes of $S_{1}$ and $S_{2}$ gradually increase with the length of T=5. The number of times $k_{M}$ that the center of the suspected target patches in the current frame falls on the corresponding concentric ring pipeline filtering is calculated. Meanwhile, the mean gray and target size have a good match. If $k_{M}$ exceeds the set threshold U=3, the centers of the suspected target patches are determined as real candidate target regions; otherwise, they are determined as false targets and rejected.

The pipeline filtering is updated according to the principle of First-In-First-Out (FIFO), and the frame that comes in first will be removed first in the update process. Then, the candidate small infrared target regions can be obtained and denoted as $R_{V}$.

### 3.3. Decision Feedback Equalization

For the top-down active attention module, Gaussian shape features are adopted to implement feature extraction, and quaternion cosine transform is used to achieve multi-dimensional fusion of Gaussian shape features. Through numerous experiments, it is found that a large number of Gaussian noises will be retained, affecting the detection of the small infrared targets. The square operation and lower weight can significantly suppress the background Gaussian-like noises. After many experiments, this paper sets the weight to 0.7 for the top-down active attention module. For the bottom-up passive attention module, the local contrast characteristic has been proven to work well in many situations and can achieve target enhancement, and the weight is set to 1. Meanwhile, motion characteristic is also important, and it helps to find small infrared targets in complex scenes. After many experiments, the weight is set to 1.3.

Based on the experimental results, feedback equalization processing is conducted to obtain the optimal weight coefficient and expression:

(22) $$F=0.7*R_{a}+S’+1.3*R_{V}$$

The final candidate regions are regarded as the real target regions.

### 3.4. Algorithm Analysis of Optimal Fast Local Contrast Algorithm

In the optimal fast local contrast algorithm, the multiscale LOG filter is used to estimate the optimal scale of the infrared small target, and box filter acceleration and structure optimization are used to enhance the computation speed. The primary focus of the algorithm analysis is the detection performance of the optimal fast local contrast algorithm to improve target detection while suppressing the background. Four cases need to be analyzed.

(23) $$d_{i}(x,y,\sigma_{i})=\frac{(m_{0}^{\sigma_{i}}-m_{Bi}^{\sigma_{i}}{}_{i})m_{0}^{\sigma_{i}}}{m_{Bi}^{\sigma_{i}}{}_{i}}(i=1,2,\ldots ,4)$$

(24) $$S(x,y,\sigma_{i})=\min\{d_{1}*d_{3},d_{2}*d_{4}\}$$

(1) When the image pixel f(x,y) belongs to the target region, then $m_{0}^{\sigma_{i}}>m_{Bi}^{\sigma_{i}}{}_{i}$,$\frac{m_{0}^{\sigma_{i}}}{m_{Bi}^{\sigma_{i}}{}_{i}}>1$, and $d_{i}>m_{0}^{\sigma_{i}}-m_{Bi}^{\sigma_{i}}{}_{i}>0$; finally, $S(x,y,\sigma_{i})>0$.
(2) When the image pixel f(x,y) belongs to the background region, then $m_{0}^{\sigma_{i}}-m_{Bi}^{\sigma_{i}}{}_{i}\approx 0$, $\frac{m_{0}^{\sigma_{i}}}{m_{Bi}^{\sigma_{i}}{}_{i}}\approx 1$, and $d_{i}=m_{0}^{\sigma_{i}}-m_{Bi}^{\sigma_{i}}{}_{i}\approx 0$; finally, $S(x,y,\sigma_{i})\approx 0$.
(3) When the image pixel f(x,y) belongs to the strong edge region, for one direction, $m_{0}^{\sigma_{i}}>m_{Bi}^{\sigma_{i}}{}_{i}$, $\frac{m_{0}^{\sigma_{i}}}{m_{Bi}^{\sigma_{i}}{}_{i}}>1$, and $d_{i}>m_{0}^{\sigma_{i}}-m_{Bi}^{\sigma_{i}}{}_{i}>0$; for the other direction, $m_{0}^{\sigma_{i}}-m_{Bi}^{\sigma_{i}}{}_{i}\approx 0$, $\frac{m_{0}^{\sigma_{i}}}{m_{Bi}^{\sigma_{i}}{}_{i}}\approx 1$, and $d_{i}=m_{0}^{\sigma_{i}}-m_{Bi}^{\sigma_{i}}{}_{i}\approx 0$; finally, $S(x,y,\sigma_{i})\approx 0$.
(4) When the image pixel f(x,y) belongs to the noise region, because it has a smaller size than the target, and its contribution to the mean gray value is limited, then $S(x,y,\sigma_{i})>S{\left(x,y,\sigma_{i}\right)}_{nosie}>0$.

In summary, the target region and the background clutter region (background, strong edge, and noise region) can be distinguished by defining the contrast by the difference and the ratio, and the back-ground clutter region is suppressed so that the background clutter region tends to be 0, thereby achieving a better effect of infrared target detection.

## 4. Experimental Results

**Figure 9:**
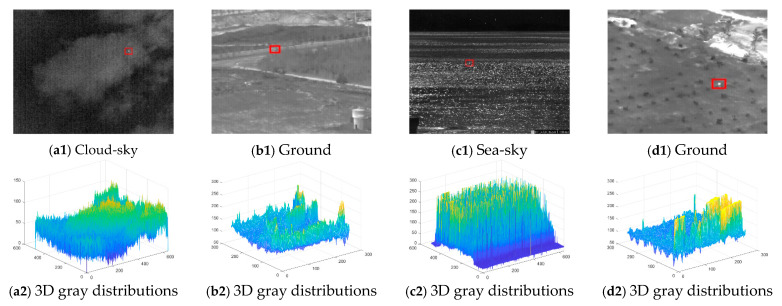
Representative infrared images and their 3D gray distributions.

Experiments were conducted in this section to verify the effectiveness of the proposed method for detecting small targets. The real infrared images are taken by infrared thermography. To investigate the performance of the proposed method in different scenes, four infrared image sequences with complex background clutters were chosen as test sequences. Figure 9 shows the target labeled by a red box in each image, while Table 1 lists the specifics of the four sequences. The background types of these sequences include sea, sky, and ground clutters. In Figure 9(a1), the target is submerged in the cloud, and many noises are randomly distributed in the whole infrared image. In Figure 9(b1,d1), plants and roads have high intensity, and the small infrared target will be blurred and easily confused with the background clutters. In Figure 9(c1), the glint will interfere with the target detection process. The algorithm’s ability to detect small infrared targets is evaluated using current metrics both from single-frame and multi-frame methods. The program is executed in Matlab2021a on a personal computer equipped with an Intel^®^ CoreTM i7-11700 CPU @ 2.50 GHz and 32 GB memory.

### 4.1. Evaluation Metrics

Different evaluation metrics are introduced for single-frame and multi-frame small infrared target detection.

The performance of target detection algorithms in single-frame recognition is often evaluated with the SCRG and BSF. The SCRG is an evaluation metric to describe the significance of the enhancement of the target after the process of an algorithm, and BSF reveals the significance of suppression of the background after the process of an algorithm. The SCRG and BSF are defined as

(25) $$SCR=\frac{G_{mt}-G_{mb}}{\sigma_{b}},\;SCRG=\frac{SCR_{out}}{SCR_{in}},\;BSF=\frac{{\left(\sigma_{c}\right)}_{in}}{{\left(\sigma_{c}\right)}_{out}}$$

The average gray of the target area is denoted as $G_{mt}$, while the average gray and standard variation of the local background area are denoted as $G_{mb}$ and $\sigma_{b}$, respectively. The signal-to-clutter ratio of the input and output images is denoted as $SCR_{out}$ and $SCR_{in}$ respectively, while the standard deviation of the input and output images is represented as ${\left(\sigma_{c}\right)}_{in}$ and ${\left(\sigma_{c}\right)}_{out}$ respectively. Additionally, the length ratio of the local background edge to the target region edge is 1.4.

To determine how well multi-frame target recognition performs, the Receiver Operation Characteristic (ROC) curve is utilized. The ROC curve plots the false positive rate (FPR) on the horizontal axis, which is the ratio of the number of false targets detected to the number of real targets, and it is also known as the precision. The true positive rate (TPR) is plotted on the vertical axis, which represents the likelihood that a detected true target is an actual true target, and it is known as the false-alarm ratio. The algorithm achieves optimal detection results when TPR is high and FPR is low, simultaneously indicating a high detection rate and low false-alarm rate. TPR and FPR are mathematically expressed as:

(26) $$\left\{\begin{array}{l} \mathrm{TPR}=\frac{\mathrm{number}\;\mathrm{of}\;\mathrm{true}\;\mathrm{target}\;\mathrm{detected}}{\mathrm{number}\;\mathrm{of}\;\mathrm{real}\;\mathrm{targets}}\\ \mathrm{FPR}=\frac{\mathrm{number}\;\mathrm{of}\;\mathrm{false}\;\mathrm{targets}\;\mathrm{detected}}{\mathrm{number}\;\mathrm{of}\;\mathrm{real}\;\mathrm{targets}} \end{array}\right.$$

There exists a relationship between the TPR and FPR. Typically, when the FPR is high, the TPR is also high. To assess the TPR and FPR while identifying the true number of targets, the LABELIMAGE2020 software is utilized to label the ground truth of small infrared targets in actual infrared images. To achieve correct detection, the Euclidean distance between the labeled targets and detection results should be less than 16 pixels. However, there could be an error in determining the actual position of the labeled targets. After conducting numerous experiments, a threshold of 16 pixels is set up.

### 4.2. Effectiveness Analysis for Single-Frame Detection

**Figure 10:**
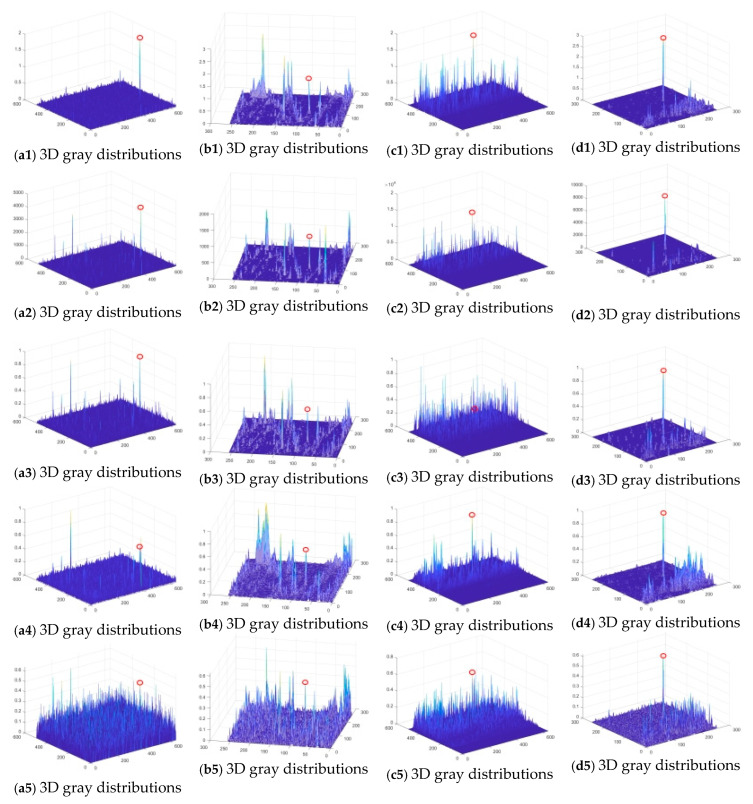
The 3D gray distributions of representative infrared images using five methods (the proposed method, ADMD, MPCM, PQFT, and WASPCM).

The effectiveness of single-frame detection is evaluated using SCRG and BSF. When the proposed method achieves high values of both SCRG and BSF, it indicates that the target has been enhanced and the background has been suppressed, making it easier to locate the target. Our proposed method was tested with about 900 infrared images to evaluate its effectiveness. Here, four representative infrared images are taken as an example, as shown in Figure 9(a1–d1). Their 3D gray distributions are shown in Figure 9(a2–d2). After the execution of the proposed method, the target is enhanced, and the clutter is suppressed, making the target easy to detect, as shown in Figure 10(a1–d1). It is noteworthy that the proposed method failed to enhance the target significantly for the image from Seq.2, as shown in Figure 10(b1). However, after using the motion information, the proposed method can enhance the target significantly and suppress the background clutters, and the target is found.

### 4.3. Comparison Analysis for Single-Frame Detection

Figure 10, and Table 2 and Table 3 present the comparison of ADMD, MPCM, PQFT, and WASPCM with our proposed method, to demonstrate its efficacy against intricate sea, sky, and ground clutters.

Figure 10 shows the 3D gray distributions obtained when applying ADMD, MPCM, PQFT, and WASPCM. These methods enhance the target while suppressing clutter. Our proposed method outperforms the other methods attributed to its use of Gaussian, contrast, and motion features for target detection, which significantly enhances the target and suppresses the background clutter. For instance, the representative infrared image chosen from Seq.2 has a lot of background clutters, which introduces many false targets. Our proposed method relies on motion characteristics and can obtain real targets. However, ADMD, MPCM, PQFT, and WASPCM cannot accurately find real targets. Obviously, our proposed method surpasses the other four methods, enabling efficient target detection.

To quantitatively compare our proposed method with four other methods in single-frame detection, the ensemble average SCRG (EASCRG) and ensemble average BSF (EABSF) were taken as indicators. Table 2 shows the detection results for Seq.1, Seq.2, Seq.3, and Seq.4. It can be seen that our proposed method achieves the highest ensemble average SCRG in all four sequences, outperforming the other four methods. Although the MPCM method achieves the highest ensemble average BSF, it consumes more time compared to our proposed method. Though the ADMD method has the lowest time consumption in Seq.1, Seq.2, and Seq.4, it obtains worse ensemble average SCRG and ensemble average BSF than our proposed method. Similarly, the PQFT method has the lowest time consumption in Seq.3, but it obtains lower ensemble average SCRG and ensemble average BSF than our proposed method. The WASPCM method has inferior ensemble average SCRG and ensemble average BSF and higher computational cost than our proposed method. Therefore, our proposed method achieves an optimal detection effect in small infrared target detection, especially under the presence of complex sea, sky, and ground clutters.

Meanwhile, an ablation experiment was conducted to demonstrate that the use of each characteristic (Gaussian, contrast, and motion characteristics) in our proposed method helps to enhance the target and suppress the background clutters. The test results of all four sequences are listed in Table 3, and the evaluation metric is ensemble average SCRG. It can be observed that our proposed method with only Gaussian characteristics obtained the lowest performance. Both the contrast and motion characteristics can improve performance. The combination of different characteristics contributes to the best performance with a maximum value of 27.6.

**Figure 11:**
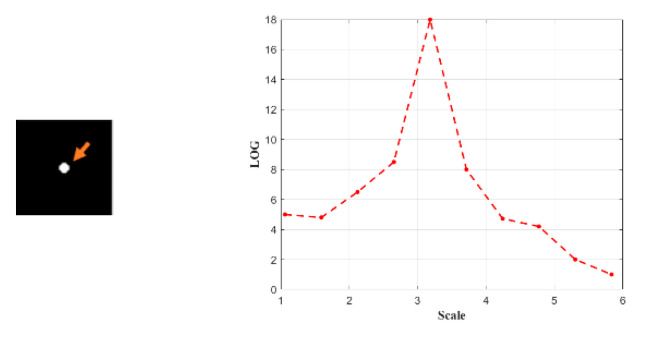
The multiscale Laplace with a scale-space constant 3.19.

Finally, the sub-window scale of the corresponding image pixel is obtained using the multiscale LOG filter. According to Figure 11, if a simulative small target has a diameter of 7, the multiscale Laplace with a scale-space constant of 3.19 will yield the maximum value. Although calculating multi-scale patches to select the most suitable scale for the target and reduce background clutter is time-consuming, the computation speed can be increased with parallel computations on GPU. To meet the real-time requirement, the algorithm speed can be further enhanced.

### 4.4. Effectiveness Analysis for Multi-Frame Detection

**Figure 12:**
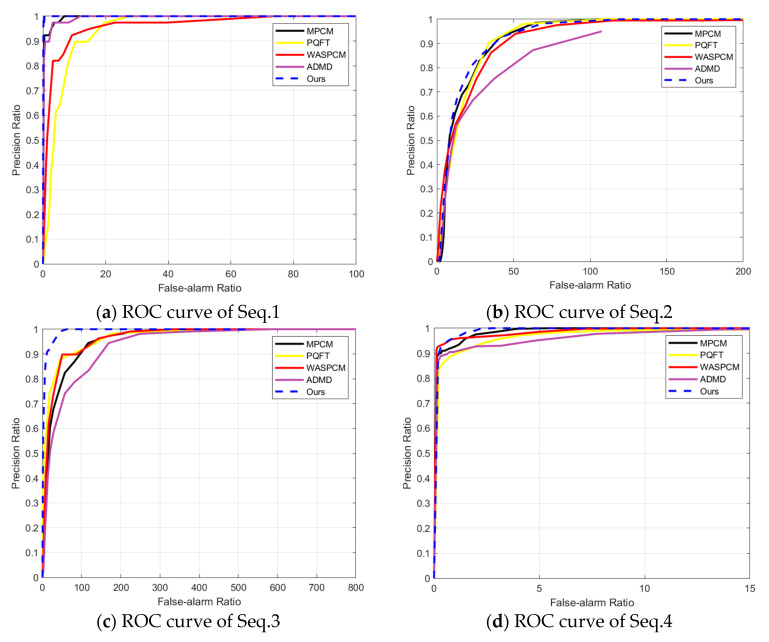
The ROC curve of four entire sequences.

To evaluate the performance of multi-frame target recognition, the TPR and FPR, also known as the precision and false-alarm ratio, are utilized to form the ROC curve. The algorithm’s performance is optimal when the precision reaches the maximum under the same FPR. Figure 12 displays the ROC curve of different methods for the entire sequence, including Seq.1, Seq.2, Seq.3, and Seq.4. Our proposed method achieves the highest precision and shows the best detection performance for the entire sequence at the same FPR. The ADMD method has the lowest time consumption in Seq.2 and Seq.4, but it obtains the worst detection performance. The PQFT method and WASPCM method can reach intermediate detection performance to find small infrared targets. The MPCM method, because of its best background suppression effect, can obtain good detection results in most cases. However, its performance is not as good as our proposed method.

Our method was evaluated with qualitative and quantitative analysis methods in this study, and it achieves the highest level of target significance and effectively reduces background clutter, thereby achieving the best detection performance.

It should be noted that, in the capture phase, accurately detecting small infrared targets is important because this is the basis of tracking in military applications. Therefore, it is crucial to study small infrared target detection.

## 5. Conclusions

This paper proposes an infrared small target detection method based on an attention mechanism against complex sea, sky, and ground clutters. Firstly, the top-down active attention module obtains knowledge-experience Gaussian shape features and achieves a complementary fusion of different feature information. Then, an optimal fast local contrast algorithm and improved circular pipeline filtering are adopted to design the bottom-up passive attention module, and the difference in contrast and motion features between the target and the back-ground is found. Finally, decision feedback equalization is adopted to fuse the results of the top-down active attention module and the bottom-up passive attention module, thereby detecting real targets, and this is useful in different scenarios to better adapt to environmental changes. Experiments were conducted on actual infrared images with intricate backgrounds involving sea, sky, and ground clutters, and experimental results reveal that the proposed method can proficiently identify targets and deliver exceptional detection results compared to other methods such as ADMD, MPCM, PQFT, and WASPCM. In particular, the best detection result is obtained in the sea background.

## Figures and Tables

**Table 1:** The specifics of four real infrared image sequences.

Background Type	Sequence Number	Frame	Target Number	Target-Size Range	Target SCR Range
Cloud-sky	Seq.1	195	1	3 × 3–5 × 5	3.16–4.84
Ground	Seq.2	144	1	4 × 4	1.42–7.74
Sea-sky	Seq.3	108	1	3 × 3–5 × 5	2.42–8.77
Ground	Seq.4	399	1	4 × 4	1.42–30.46

**Table 2:** EASCRG, EABSF, and the computational cost of the proposed method and four baseline methods for Seq.1, Seq.2, Seq.3, and Seq.4.

		Index	Seq.1	Seq.2	Seq.3	Seq.4
	Metrics					
Proposed	EASCRG		30.5	2.89	12.5	66.2
	EABSF		2099.5	645	1129.6	1444.4
	Time(s)		0.6027	0.1379	1.1598	0.1316
ADMD [31]	EASCRG		26.9726	83.6801	11.8651	47.0298
	EABSF		0.6574	0.4434	0.0977	0.3810
	Time(s)		0.2083	0.0351	0.9446	0.0298
MPCM [29]	EASCRG		16.7465	2.8000	8.0934	28.8780
	EABSF		3077.1	786.6690	1684.5	2563.3
	Time(s)		0.8113	0.1489	1.7263	0.1361
PQFT [34]	EASCRG		4.3851	0.7913	2.1392	13.6803
	EABSF		1434.04	676.3588	850.3719	1398.2.
	Time(s)		0.3138	0.0360	0.3026	0.3076
WASPCM [36]	EASCRG		4.2918	0.8718	5.35	7.2413
	EABSF		1371.1	663.5638	1667.3	1831.1
	Time(s)		1.3857	0.1674	0.8887	0.1383

**Table 3:** The ensemble average SCRG with combination of different characteristics.

Each Characteristic	Ensemble Average SCRG
Gaussian	7.5
Gaussian + motion	9.9
Gaussian + contrast	11.7
Gaussian + contrast + motion	12.9

## Data Availability

Not applicable.
